# Antidepressant Flavonoids and Their Relationship with Oxidative Stress

**DOI:** 10.1155/2017/5762172

**Published:** 2017-12-19

**Authors:** Lucian Hritcu, Radu Ionita, Paula Alexandra Postu, Girish Kumar Gupta, Hasan Turkez, Tamires Cardoso Lima, Caroline Uchôa Souza Carvalho, Damião Pergentino de Sousa

**Affiliations:** ^1^Department of Biology, Alexandru Ioan Cuza University of Iasi, Bd. Carol I No. 11, 700506 Iasi, Romania; ^2^Department of Pharmaceutical Chemistry, M. M. College of Pharmacy, Mullana, Maharishi Markandeshwar University, Ambala, Haryana 133203, India; ^3^Department of Molecular Biology and Genetics, Erzurum Technical University, 25000 Erzurum, Turkey; ^4^Departamento de Farmácia, Universidade Federal de Sergipe, 49100-000 São Cristóvão, SE, Brazil; ^5^Departamento de Ciências Farmacêuticas, Universidade Federal da Paraíba, 58051-970 João Pessoa, PB, Brazil

## Abstract

Depression is a serious disorder that affects hundreds of millions of people around the world and causes poor quality of life, problem behaviors, and limitations in activities of daily living. Therefore, the search for new therapeutic options is of high interest and growth. Research on the relationship between depression and oxidative stress has shown important biochemical aspects in the development of this disease. Flavonoids are a class of natural products that exhibit several pharmacological properties, including antidepressant-like activity, and affects various physiological and biochemical functions in the body. Studies show the clinical potential of antioxidant flavonoids in treating depressive disorders and strongly suggest that these natural products are interesting prototype compounds in the study of new antidepressant drugs. So, this review will summarize the chemical and pharmacological perspectives related to the discovery of flavonoids with antidepressant activity. The mechanisms of action of these compounds are also discussed, including their actions on oxidative stress relating to depression.

## 1. Introduction

Besides cognitive deficits, Alzheimer's disease (AD) is characterized by noncognitive features which are the behavioral and psychological symptoms of dementia (BPSD) [1]. Of all the BPSD, the prevalence of depression is the most noteworthy, at 40% [2], and could be considered to be a risk factor for AD [3]. The neurotoxic effects of depression include atrophy of hippocampus linked to over secretion of cortisol or abnormally low concentration of brain-derived neurotrophic factor (BDNF) [4]. Furthermore, it has been recommended that depression could be induced by metabolic disorders of monoamine neurotransmitters that are engaged in noradrenaline (NE), serotonin (5-HT), and dopamine (DA) signaling [5]. Anxiety and depression have been shown to increase the severity of cognitive decline in AD patients [6]. Furthermore, anxiety is more common in individuals with dementia than in individuals without dementia [7] and it is associated with worse quality of life, problem behaviors, impediments in activities of daily living, nighttime awakenings, and poorer neuropsychological performance, even after controlling for depression [8]. The World Health Organization (WHO) estimates that around 350 million individuals suffer from depression and anticipates that by 2020 the disorder will be the second driving reason of disability worldwide. As of now, there are numerous effective antidepressants available, yet a few antidepressants caused insufficient and unsatisfactory results in around 33% of all subjects treated [9–11]. Along these lines, endeavors ought to be sought after for the development of the newer antidepressant agents with better efficacy and fewer side effects.

The different forms of monoamine oxidases (MAO-A and MAO-B) were considered as relevant for key events in intrinsic cell death pathways, particularly those focused on oxidative stress and peroxyradical-mediated mechanisms via causing to the production of hydrogen peroxide as a byproduct of the reaction between the MAOs (monoamine oxidases) and their monoamine substrates [12]. Especially the upregulation of MAO-A prompted increments of 5-hydroxyindoleacetic acid/5-HT ratio, oxidative stress, leading to nuclear factor-*κ*B (NF-*κ*B) activation, inflammation, and apoptosis [13, 14]. The patients with chronic neurodegenerative symptoms like depression and apathy are for the most part treated with drugs that elevate biogenic amine levels. This common therapeutic strategy was believed to be responsible for the generation of neurotoxic aldehydes and enhanced oxidative stress which in turn further increases the biogenic amine turnover. The exploratory examinations likewise uncovered this last condition influencing and accelerating the course of neurodegeneration. Truth be told, the *in vivo* findings from chronic, unpredictable stress-induced depression models of mice prompted that the depression formation was strongly emphatically connected with the increased activities of MAOs and malondialdehyde (MDA) amounts and diminished glutathione levels, glutathione reductase, and glutathione peroxidase activities in the brain [15–17]. In a current report by Czarny et al. [18], it was accounted that elevated levels of reactive oxygen and nitrogen species (ROS and RNS) caused oxidative DNA damage in depressed patients. It is well known that chronic oxidative stress due to ROS and RNS production has a huge potential to drive carcinogenesis by altering the expression of cancer-related genes causing mutation and transformation. Concordantly, it was discovered that gastric cancer patients with depression are under elevated levels of oxidative stress conditions that are accompanied by the dysfunction of numerous protooncogenes [19, 20].

Herbal therapies may be a fascinating and successful option in depression treatment, since a large number of herbal preparations have demonstrated psychotherapeutic activities. The search for new pharmacotherapy from medicinal herbs and constituents isolated from plant extracts for psychiatric disorders, including depression, has advanced expressively over the previous decade [21]. For instance, a flavonoid-rich fraction obtained from the seed extract of *Monodora tenuifolia* was able to do altogether to decrease behavioral alterations in forced-swim stressed rats and in addition exert protective effects against induced oxidative stress, supporting its antidepressant effect [22]. In another investigation, the methanolic extract from the species *Byrsonima crassifolia* (L.) Kunth (Malpighiaceae) uncovered antidepressant activity in the forced swimming test and the antioxidant flavonoids rutin, quercetin, and hesperidin perhaps are engaged with the antidepressant effects of *B. crassifolia* (L.) Kunth [23–26]. Flavonoids are a broad class of secondary metabolites copious in plants and different foods. They have been distinguished in an assortment of a variety of fruits and vegetables and confer color, flavor, and aroma, as well as nutritional and health benefits. Polyphenol flavonoids are the most effective functional ingredients with biological activities. Many flavonoids possess antioxidant and antidepressant activities [10, 24, 25]. It is widely reported that oxidative stress assumes a critical part in the development of various diseases [27], including psychopharmacological disorders [28]. Indeed, the connection between oxidative stress and depression has been studied and discussed in some reviews [28–31]. Accordingly, this review provides a detailed overview of the current state of knowledge about the antidepressant activities of flavonoids, as well as their relationship with oxidative stress.

## 2. Methodology

The search was conducted in the scientific database PubMed, focusing on works published during the last six years (January 2011 to December 2016). The data were selected using the following terms: “flavonoid” and “antidepressant” as well as the names of experimental models of depression in animals such as “Forced Swim Test” and “Tail Suspension Test” and “Oxidative Stress.”

## 3. Results and Discussion

### 3.1. Flavonoids and BDNF Expression

BDNF is a neurotrophin expressed in the brain and participates in a range of intracellular signaling processes, neuronal protection and survival, axonal and dendritic morphology and synaptic plasticity [32]. It has been documented that BDNF is involved in a number of neuropsychiatric disorders such as affective disorders, schizophrenia, addiction, eating disorders, and neurodevelopmental disorders [33]. Decreased levels of BDNF are among the most frequently validated biomarkers of depressive disorder [32]. Importantly, reduced BDNF levels have been reported in postmortem brain samples from AD patients [34].

*Hesperidin* (1), a natural flavanone glycoside predominant in citrus fruits, has been accounted with useful therapeutic properties such as antidiabetic [35], antioxidant [36], neuroprotective [37], and anticancer [38]. El-Marasy et al. [39] reported the antidepressant effect of hesperidin in streptozotocin-induced diabetic rats (Table 1). The outcome of the study indicated that the effects of hesperidin are mediated at least in part, via its modulatory effect on hyperglycemia, its antioxidant and anti-inflammatory activities, alteration of BDNF levels, and activation of the brain's monoaminergic system. Furthermore, Donato et al. [40] observed that chronic administration of hesperidin resulted in an increase in hippocampal BDNF levels. These authors concluded that the antidepressant effect of hesperidin is mediated by inhibition of L-arginine-NO-cGMP pathway and by an increase of the BDNF levels in the hippocampus. In another examination, Li et al. [41] explored the antidepressant-like mechanism of hesperidin in mice exposed to chronic mild stress (CMS). The obtained results showed the ability of hesperidin to ameliorate the reduction of sucrose preference and reverse the augmented immobility time induced by CMS. All these information endorse the antidepressant effect of hesperidin and suggest that extracellular signal-regulated kinase- (ERK-) BDNF signaling pathway is involved in the antidepressant-like activity of this flavanone.

*Chrysin* (2), a natural flavonoid predominant in bee propolis, honey, and several plants, possesses multiple biological activities such as anti-inflammatory, antineoplastic, hypolipidemic, and antioxidant [42–44]. In addition, Filho et al. [45] revealed the antidepressant effect of chrysin in mice subjected to chronic unpredictable mild stress (Table 1). The authors proposed that upregulation of BDNF levels in the hippocampus and prefrontal cortex of stressed mice may be associated with the antidepressant effects of chrysin. In another study done by the same research group members [46], they showed that the treatment with chrysin caused the attenuation of depressive-like behavior and hippocampal changes in olfactory bulbectomized mice, reinforcing that BDNF plays an important role in the antidepressant effect of this flavonoid. Further, Filho et al. [47] likewise analyzed the neurochemical parameters correlated with the antidepressant property of chrysin in mice exposed to unpredictable chronic stress. The authors suggested an association existing between the antidepressant-like action of chrysin and the proinflammatory cytokines synthesis, 5-hydroxytryptamine metabolism, kynurenine pathway, and caspases activities.

*Naringenin* (3), a dietary flavonoid prevalent in the peels of citrus fruit, has various biological actions such as a cognitive enhancer [48] and inhibits monoamine oxidase activity [49] and neuroprotection [50]. Likewise, naringenin was found to display antidepressant effects [51]. The authors inferred that naringenin treatment can suppress neuroendocrine signaling and stimulate monoamines, which bring about upregulation of BDNF in the mice hippocampus.

*Astilbin* (4), a natural flavonoid heteroside displayed in the plants of *Smilax* or *Hypericum perforatum* L., has different pharmacological actions such as antioxidant, free radical scavenging, and anti-inflammatory function [52, 53]. Lv et al. [54] detailed additionally about the antidepressant effect of astilbin. They suggested that the effects of astilbin observed in experimental mice of depression are mediated by upregulation of the BDNF signal pathway and monoaminergic neurotransmitters discharge in the mice cortex.

*Icariin* (5) is a major bioactive compound from the species *Herba Epimedii* (*Epimedium brevicornum* Maxim), a traditional Chinese medicinal herb, used for centuries for treating various conditions including depression [55]. Among 19 metabolites originated of icariin, icariin has been found to possess neuroprotective potential [55]. Wu et al. [55] reported that icaritin is a novel antidepressant and partly restored social defeat-induced impairment of glucocorticoid sensitivity and hypothalamic-pituitary-adrenal (HPA) axis hyperactivity. These effects are at least partially attributed to normalization of the glucocorticoid receptor function and increases in BDNF expression. In addition, Liu et al. [56] also reported that icariin exerted an antidepressant effect in an unpredictable chronic mild stress model of depression in rats and is associated with the regulation of hippocampal neuroinflammation. In another study, Wei et al. [57] investigated the effects of icariin treatment in a model of depression in rats induced by unpredictable chronic mild stress. The obtained results suggest the therapeutic efficacy of icariin as a potential antidepressant. Furthermore, the antidepressant activity of this flavonoid heteroside occurs via different targets in both the hippocampus and prefrontal cortex.

*7,8-Dihydroxyflavone* (6) acts as a TrkB receptor-specific agonist and can mimic BDNF action. Also, it demonstrated therapeutic efficacy in animal models of various neurological diseases [58] such as Parkinson's disease, stroke [59], and depression [60]. Liu et al. [58] reported that 7,8-dihydroxyflavone can penetrate the brain-blood barrier (BBB) and mimics BDNF action. Also, the authors reported that this compound poses more prominent physiological activities than other reported peptide mimetics or small molecules, supporting the fact that 7,8-dihydroxyflavone is a superior compound with oral bioavailability for TrkB agonist drug development.

*Hyperoside* (7) is a natural flavonoid isolated from *Apocynum venetum* L. leaves [61]. Zheng et al. [61] reported that hyperoside possesses antidepressant effects via cytoprotective action related to the elevation of the expression of BDNF and CREB through the signal pathway AC-cAMP-CREB within the PC12 cell line. In addition, Haas et al. [62] concluded that this flavonoid heteroside, extracted from the crude extract of *Hypericum caprifoliatum* Cham. & Schltdl. (Guttiferae), presented a depressing effect on the central nervous system (CNS) and either an antidepressant effect in rodents mediated by the activation of D2-DA receptors.

*Baicalein* (8) is one of the most active flavonoids found in the dry roots of *Scutellaria baicalensis* Georgi. It has been reported that baicalein can get across the BBB [63]. Also, various studies have indicated that baicalein has proved to be a superior free radical scavenger and xanthine oxidase inhibitor [63, 64]. Xiong et al. [65] reported that this flavone exhibited antidepressant effects. In addition, baicalein reversed the reduction of ERK phosphorylation and the level of BDNF expression in the hippocampus of chronic mild stress model rats. These results suggest that baicalein produces an antidepressant-like effect, and this effect is at least partly mediated by hippocampal ERK-mediated neurotrophic action. Furthermore, Li et al. [66] suggested that baicalein could prevent the chronic mild stress-induced depressive-like behavior through the inhibition of cyclooxygenase-2 in rat brain and subsequently resulted in a reduction of prostaglandin E_2_ levels in the brain.

*3,5,6,7,8,3*′*,4*′*-Heptamethoxyflavone* (9) is a polymethoxyflavone found in several citrus fruits [67]. This polymethoxyflavone possesses several biological activities, including anti-inflammatory, neuroprotective, [68] and the immunomodulatory function [67]. In a study performed by Sawamoto et al. [69], these authors suggested that the 3,5,6,7,8,3′,4′-heptamethoxyflavone exerts antidepressant activity by inducing the expression of BDNF. This flavone improved corticosterone-induced depression-like behavior and repaired BDNF expression, neurogenesis, and neuroplasticity in the hippocampus.

### 3.2. Flavonoids and Monoaminergic Systems

The monoamine theory of depression states that depression is associated with a decrease in monoamine levels in the synaptic cleft, namely, of the catecholamine NE and of the indoleamine 5-HT [70]. The main biochemical causes of depression are metabolic disorders of monoamine neurotransmitters that are involved in NE, 5-HT, and DA signaling [5, 10]. Moreover, in many depressed patients, the impairment of the function of the HPA axis was noticed [71]. It has been reported that many flavonoids possess antioxidant, anti-inflammatory, and antidepressant activities in animal studies [72, 73].

*Kaempferitrin* (10) is the main secondary metabolite extracted from the *Justicia spicigera* Schltdl. (Asteraceae) plant. It has been documented that this plant is used for its analgesic, antidiabetic, anti-inflammatory, and antiseizure potential, as well as a tonic [74]. Cassani et al. [74] reported that kaempferitrin exhibited antidepressant effects in two behavior models in mice. In addition, its effect could be related to serotonergic neurotransmitter system action, mainly through its interaction with presynaptic 5-HT_1A_ receptors. Also, the authors suggested the involvement of the HPA axis in the antidepressant-like effect of kaempferitrin.

The antidepressant effect of the *Hemerocallis citrina* Baroni is mediated by the contributions of flavonoids, especially *rutin* (11) and hesperidin (1) [75]. Its antidepressant effects are due to the interaction with serotonergic, noradrenergic, and dopaminergic systems [76]. The antidepressant effect of hesperidin depends on its interaction with serotonergic 5-HT_1A_ receptors [77]. The aforementioned mechanism of hesperidin action is also supported by Souza et al. [77] studies by interaction with the serotonergic 5-HT_1A_ receptors. Filho et al. [78] also reported the antidepressant effect of hesperidin in a mice model of anxiety, through its interaction with *κ*-opioid receptors, but not with the *δ*-opioid, *μ*-opioid, or adenosinergic receptors.

*Luteolin* (12) is a common flavonoid with various pharmacological actions such as antioxidant, anticancer, memory-enhancing, and anxiolytic, indicating that luteolin could easily penetrate the BBB [79]. De la Peña et al. [80] reported that luteolin mediates the antidepressant effects of *Cirsium japonicum* Fisch. ex DC., possibly by potentiation of the GABA_A_ receptor-Cl-ion channel complex. Also, Ishisaka et al. [79] have shown that luteolin attenuated the expression of endoplasmic reticulum stress-related proteins in the hippocampus of corticosterone-treated depression model mice.

*Vitexin* (13) is a flavone glycoside present in foodstuffs and nutraceuticals [81]. It has been shown that vitexin has multiple pharmacological effects such as inhibitory effects on adipogenesis [82], platelet aggregation [83], *α*-glucosidase [84] and urease [85], and antitumor/antimetastatic [86], antioxidant [87], anti-inflammatory, [88] and peripheral analgesic [89] activities. Among plants, *Passiflora incarnata* L. (Passifloraceae) have been found to be the main source of vitexin, with significant effects on the CNS, including anxiolytic effects. Can et al. [81] reported that vitexin possess antidepressant effect mediated by an increase in catecholamine levels in the synaptic cleft as well as by interactions with the serotonergic 5-HT_1A_, noradrenergic *α*_2_, and dopaminergic D_1_, D_2_, and D_3_ receptors.

*Amentoflavone* (14) is a natural flavonoid with many biological properties such as antioxidative, anti-inflammatory, and neuroprotective effects [90]. Ishola et al. [91] reported the antidepressant and anxiolytic effects of amentoflavone isolated from *Cnestis ferruginea* Vahl ex DC. in mice. The authors concluded that amentoflavone produces its antidepressant effects through interactions with the 5-HT_2_ receptor and *α*1- and *α*2-adrenoceptors while the anxiolytic effect involved the ionotropic GABA receptor.

Naringenin (3) is a flavanone found in high amounts in the peels of citrus fruits with several biological effects such as neuroprotective [92] and monoamine oxidase inhibitory activity [34]. Moreover, naringenin exhibited antidepressant effects via monoaminergic systems [93]. Yi et al. [94] reported the antidepressant-like behavioral, neurochemical, and neuroendocrine effects of naringenin in the mouse repeated the tail suspension test. The authors concluded that the antidepressant effects of naringenin may be mediated by an interaction with neuroendocrine and neurochemical systems.

*Fisetin* (15) is a natural flavonoid found especially in strawberries and other fruits or vegetables. This flavonoid has various biological activities, including antioxidant, anti-inflammatory, and neuroprotective effects [95, 96]. Zhen et al. [97] reported that the antidepressant-like effect of fisetin involves the serotonergic and noradrenergic systems. The authors concluded that the positive effects of fisetin on the depressive response are likely mediated via the central serotonergic and noradrenergic system by inhibiting the monoamine oxidase enzyme activity. In another study, Yu et al. [98] evaluated the ability of fisetin to modulate depressive-like behavior in a lipopolysaccharide- (LPS-) induced acute neuroinflammation model. The authors concluded that fisetin is a potential candidate for clinical mental disorder therapy since it can correct depressive-like behavior in LPS-induced depression in mice model.

*Nobiletin* (16) is a dietary flavonoid abundant in the peels of citrus with many potential health benefits. It has been reported that nobiletin exerts protective effects on *β*-amyloid peptide-induced impairment of learning ability [99], improved the memory impairment, reduced the *β*-amyloid peptide levels [100], and had neuroprotective effects on ischemia-induced neuronal death in the hippocampal CA1 region [101]. Yi et al. [102] reported the involvement of monoaminergic systems in the antidepressant-like effect of nobiletin. The authors concluded that the antidepressant-like effect of nobiletin seems to be mediated by an interaction with the serotonergic (5-HT_1A_ and 5-HT_2_ receptors), noradrenergic (*α*1-adrenoceptor), and dopaminergic (D_1_ and D_2_ receptors) systems.

*Quercetin* (17) is a dietary flavonoid presented in high amount in onion, apple, broccoli, and wine, as well as plants like *Ginkgo biloba* L. and green tea [103]. It has been reported that quercetin is a powerful radical scavenger flavonol and so that it fortifies the antioxidant defense system [104]. In addition, quercetin increase 5-HT and norepinephrine availability in synaptic cleft that seems to be dysregulated in depression. Demir et al. [105] reported antidepressant-like effects of quercetin in diabetic rats (Table 1). The authors concluded that quercetin may be considered as a partially useful supplement for the treatment of diabetic depression, and the antidepressant-like properties of quercetin seem to be independent of the HPA axis. Furthermore, Scheggi et al. [106] reported antidepressant activity of *Hypericum perforatum* L. (Hypericaceae) related to the flavonoid components of this species including quercetin. Also, Rinwa and Kumar [107] have shown that quercetin suppresses the microglial neuroinflammatory response and induces the antidepressant-like effect in olfactory bulbectomized rats.

### 3.3. Antioxidant Effect of Antidepressant Flavonoids

Contemporary endeavours are being given to explore novel natural remedies for better positive effect with no or less toxic effects alternatives to conventional antidepressants. Despite not being fully studied or understood, naturally occurring flavonoids have demonstrated less or more neuroprotective activities. The neuroprotective mechanisms of antidepressant effects remain to stay vague, in spite of the fact that it is proposed that flavonoids generally exert their antidepressant-like effects via altering behavior, cytokine levels, oxidative stress, and energy metabolism parameters. In addition to antioxidative action, each flavonoid follows its idiocratical one or more different pathways from these general routes against advancement and progression of depression including prevention of mitochondrial membrane potential dissipation, agonizing GABA-benzodiazepine receptors interaction with *κ*-opioid receptors and kynurenine pathway (KP), acetylcholinesterase activity regulation, helping to maintain brain plasticity, inhibition of L-arginine-NO, extracellular signal-related kinase (ERK) 1/2 and AKT phosphorylation pathways, modulation of intracellular calcium overload and K^+^ channels, downregulation of Bax, caspases 3 and 9, and cytochrome C (Cyt-C) protein expression, and upregulation of Bcl protein expression were also afforded to positive impacts of flavonoids in the treatment depression [108–112].

**Figure 1:**
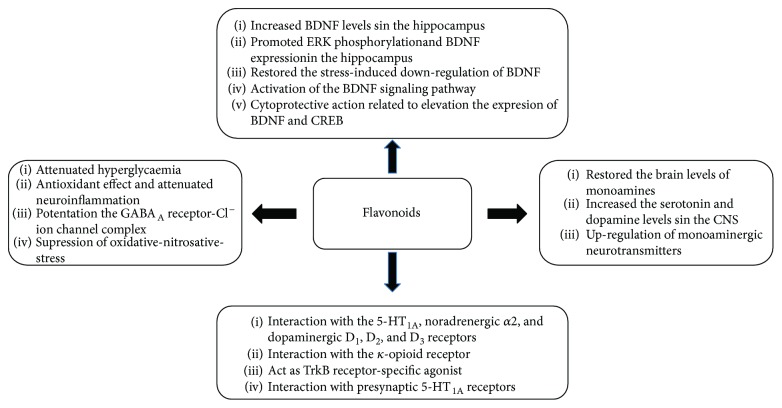
Possible mechanism of action of flavonoids with antidepressant activity.

Due to their serious side effects of the current MAO inhibitors and the urgent need for novel ones, natural products have been considered as alluring focuses for pharmacologists. Exceptionally, a few reports obviously settled higher effectiveness by flavonoids compared to placebo intake and a similar activity was observed when comparing to several antidepressant drugs. Among these flavonoids that display antidepressive-like activity such as hesperidin (1), naringenin (3), quercetin (17), and astilbin (4) have been appeared to diminish depressive symptoms in animals experimental trials or *in vitro* models (Figure 1), mainly for the most part by means of the (i) inhibiting monoamine oxidases (MAOs) and (ii) altering oxidative/antioxidant defenses and/or (iii) inflammatory responses [113–115].

Several flavonoids have been appeared to avert against neurodegenerative disorders and depressive insults. However, limited studies are recorded in the literature with respect to the neuroprotective mechanisms of these naturally occurring compounds more particularly in the treatment of depressive disorders. From the literature scanning, it was clearly comprehended that the conceivable mode of action of flavonoids included quenching free radical elements and the stimulation of internal antioxidant enzymes mainly. In fact, hesperidin led to the decrease of ROS generation, enhances of superoxide dismutase (SOD) and glutathione (GSH) levels, and reduced MDA formation in cultured different human cell lines including HaCaT and ARPE-19 cells [116–118]. While amentoflavone (14) displays inhibitory consequences on the productions of superoxide anion and total reactive oxygen species (ROS) [119] and neuroprotective activity by means of restoration of the reduced superoxide dismutase (SOD) activity, glutathione reductase (GR) activity, and glutathione content induced by glutamate [120]. Vitexin (13) is another flavonoid that shows neuroprotective action demonstrated in studies. In the mechanism of action, suppression of isoflurane-induced caspase-3 activation and increased *β*-secretase 1 levels in PC12 cells was proposed. It has also been reported to decrease the levels of isoflurane-induced cytosolic calcium and reactive oxygen species [121]. Likewise, cellular ROS production induced by several oxidative damaging agents was attenuated by pretreatment with chrysin (2) [122], naringenin (3) [123], astilbin (4) [53], icariin (5) [124], 7,8-dihydroxyflavone (6) [125], hyperoside (7) [126], baicalein (8) [127], rutin (11) [128], luteolin (12) [129], fisetin (15) [130], nobiletin (16) [131], kaempferitrin (10) [132], and quercetin (17) [133].

Another revealed the antioxidative mechanism of action of flavonoids was through the chelation of transition metal elements. Then, these natural compounds enabled metals to chelate or binds to metal ions in humans and animals to block them being accessible to oxidation [116]. Now, hesperidin (1) [36], naringenin (3) [123], astilbin (4) [134], luteolin (12) [135], and quercetin (17) [136] appeared to chelate metal ions such as iron, copper, and zinc in showing their antiradical properties. Notwithstanding free radicals scavenging and chelating of metal ions, several flavonoids, including hesperidin (1), astilbin (4), luteolin (12), baicalein (8), and quercetin (17), played key roles in inhibiting free radical generating enzymes such as myeloperoxidase, xanthine oxidase, lipoxygenase, microsomal monooxygenase, and NADPH oxidase [137–143]. The polymethoxyflavones nobiletin (16) and 3,5,6,7,8,3′,4′-heptamethoxyflavone (9), found in young fruits of *Citrus unshiu* Marc., inhibit NO production, LPS-induced iNOS protein, and mRNA expression by NF-*κ*B activation and p38-mitogen-activated protein kinase (MAPK) phosphorylation. Interestingly, the young citrus fruit demonstrated a neuroprotective effect by delaying neurodegeneration in hippocampal CA1 neurons of the Mongolian gerbil after global ischemia [144]. It is revealed that depression is closely associated with altered cellular resilience, selective structural changes, and neuronal atrophy of the hippocampus [145, 146]. Therefore, a possible reversal of these changes structures by constituents of the plant, such as antioxidant flavonoids nobiletin (16) and 3,5,6,7,8,3′,4′-heptamethoxyflavone (9), should be an interesting way to treat this behavioral disorder. Truth be told, it has as of late been exhibited that orally administered 3,5,6,7,8,3′,4′-heptamethoxyflavone (9) is beneficial for the upregulation of BDNF in the hippocampus via the ERK1/2/MAP system. These information ought to be identified with the antidepressant effects of this compound [147].

Hyperoside (7) inhibits 13-HPODE-induced ROS production in PC-12 cells. This compound is found in *Apocynum venetum* L. which likewise has antidepressant and antioxidant activity. Hyperoside (7) is one of the constituents in the extract of this plant that contribute to these activities. Whereas oxidative stress may be associated with the advancement of depression, both extract and compound **7** must have protective action against oxidative stress in nerve cells [148].

Once more, this class of compounds might act as antidepressant agents endowed with multiple mechanisms of action in the CNS, increasing central neurotransmission, limiting the reabsorption of bioamines by synaptosomes, and modulating the neuroendocrine and GABA_A_ systems [149]. Curiously, support with several flavonoids strengthened the pharmacokinetic efficacy of many medications for depression. The flavonoids hesperidin (1) and naringenin (3) enhanced the area under the curve (AUC), maximum plasma concentration (*C*_max_), and elimination half-life (*t*_1/2_) of rasagiline, a selective monoamine oxidase-B inhibitor, with a concomitant reduction in clearance (CL/F) in both single and multiple dose studies [150], while quercetin (17) affects glutamatergic neurotransmission in rat brain [151] evidencing the action of this compound in the glutamateric framework.

## 4. Conclusions

Considering that oxidative stress is unequivocally associated with the advancement of depression, the reported data suggest that the utilization of these flavonoids may help in reducing the symptoms of depression, notably via supplementation of dietary flavonoids in which they are significantly related with the minimization of depression risk due to their great antioxidative natures. Regrettably, advanced investigations are needed to fully understand the mode of action to neuroprotection, biotransformation of their metabolites in the body, and interaction properties with receptors related to depression.

## Figures and Tables

**Table 1:** Flavonoids studied in experimental depression.

Flavonoid	Administration	Animal species	Depression model	Observed effects	Mechanism of action	Observation	Reference
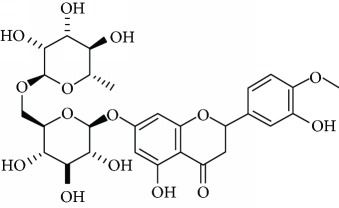 Hesperidin (1)	Oral 25, 50, or 100 mg/kg	Rat	FST	Reduced immobility time	Attenuated hyperglycaemia Increased the neurogenesis Increased the BDNF levels Restored the brain levels of monoamines	DR+	[39]
	Oral 0.4, 4, 8, 16, and 32 mg/kg (fixed-ratio combination of rutin and hesperidin 71 : 21.5 *w*/*w*)	Mice	TST, OFT	No effects	Increased the 5-HT and DA levels in the CNS	DR+	[75]
	Intraperitoneal, acute, and chronic 0.01, 0.1, 0.3, and 1 mg/kg	Mice	TST, OFT	Reduced immobility time	Increased the BDNF levels in the hippocampus Decreased the nitrate/nitrite (NOX) levels in the hippocampus	DR+	[40]
	Intraperitoneal 0.01, 0.1, 0.3, and 1 mg/kg	Mice	TST, FST, and OFT	Reduced immobility time	Interaction with the serotonergic system (5-HT_1A_ receptors) Antioxidant effect	DR+	[77]
	Intraperitoneal 0.01, 0.1, 0.3, and 1 mg/kg	Mice	FST, OFT	Reduced immobility time	Interaction with the *κ*-opioid receptor	DR+	[78]
	Oral 25 and 50 mg/Kg	ICR mice	CMS, FST	Reduced immobility time	Reversed the reduction of sucrose preference. Promoted ERK phosphorylation and BDNF expression in the hippocampus	DR+	[41]
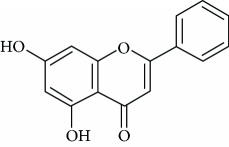 Chrysin (2)	Oral 5 or 20 mg/kg	Mice	CUMS, SPT, OFT, and FST	Reduced immobility time	Increased the sucrose preference Increased BDNF and NGF levels in the hippocampus and cortex prefrontal Antioxidant activity	DR+	[45]
	Oral 5 or 20 mg/kg	Mice	ST, OFT, and FST	Reduced immobility time	Increased BDNF levels Modulation of cytokines levels	DR+	[46]
	Oral 5 or 20 mg/kg	Mice	ST, rota rod, and TST	Reduced immobility time	Decreased 5-HT levels in the hippocampus Reduced TNF-*α*, IL-1*β*, IL-6, and kynurenine levels Increased caspases activities in cerebral structures	DR+	[47]
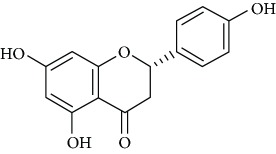 Naringenin (3)	Oral 5, 10 or 20 mg/Kg	ICR mice	OFT, TST	Reduced immobility time	Increased 5-HT and norepinephrine and GR levels	DR+	[94]
	Oral 20 mg/Kg	ICR mice	CUMS, SPT, and NSFT		Enhanced the BDNF expression in the hippocampus but not in the frontal cortex Restored the stress-induced downregulation of BDNF	DR+	[51]
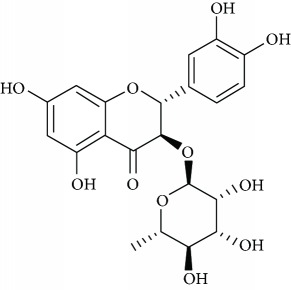 Astilbin (4)	Intraperitonial 10, 20, or 40 mg/kg	Mice	CUMS, OFT, FST, TST, and SPT	Reduced immobility time	Upregulation of monoaminergic neurotransmitters (5-HT and DA) Activation of the BDNF signaling pathway	DR+	[54]
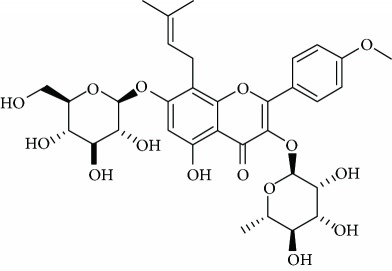 Icariin (5)	Oral 20 or 40 mg/Kg	Rat	SPT, CMS, and FST	Reduced immobility time	Attenuated oxidative stress damage and neuroinflammation Inhibited the NF-*κ*B signaling pathway and NLRP3-inflammasome activation	DR+	[56]
	Oral 20 mg/Kg	Mice	Social defeat		Attenuated the increases in serum IL-6 and TNF-*α* level Restored social defeat-induced impairment of glucocorticoid sensitivity	DR+	[55]
	Oral 20 or 40 mg/Kg	Rat	SPT		Attenuated the development of depression-like behaviors Reversed the upregulated expression of nuclear GR in the prefrontal cortex	DR+	[57]
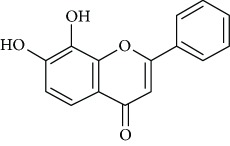 7,8-Dihydroxyflavone (6)	Oral 5 mg/Kg	Mice	FST, TST	Reduced immobility time	Acts as a TrkB receptor-specific agonist Can penetrate the BBB and mimics BDNF action		[58]
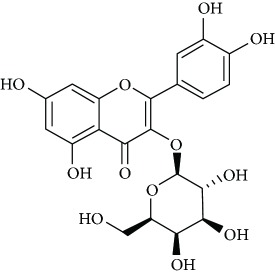 Hyperoside (**7**)	*In vitro* 2.5, 5, and 10 *μ*g/mL	PC12 cell line	Evaluation of cell viability		Protected PC12 cells from the lesion induced by corticosterone Cytoprotective action related to elevation the expression of BDNF and CREB	DR+	[61]
	Intraperitonial 10, 20, or 40 mg/Kg	Mice	FST	Reduced motor activity	Activation of D2-DA receptors	DR+	[62]
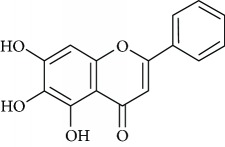 Baicalein (8)	Oral 10, 20, or 40 mg/Kg	Rat	CMS	Reduced immobility time	Decreased the activity and expression COX-2 Attenuated the reduction of sucrose preference Reduced the PGE_2_ levels in the brain	DR+	[5]
	Intraperitonial 1, 2, or 4 mg/Kg	Mice/ rat	OFT, FST, and TST	Reduced immobility time	Reversed the reduction of extracellular ERKs phosphorylation Enhanced the level of BDNF expression in the hippocampus	DR+	[65]
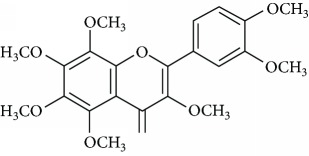 3,5,6,7,8,3′,4′-Heptamethoxyflavone (9)	Subcutaneous 50 mg/Kg	Mice	FST, TST	Reduced immobility time	Attenuated corticosterone-induced depressive-like behavior Induced the expression of BNDF in hippocampus Enhanced the neurogenesis and neuroplasticity		[69]
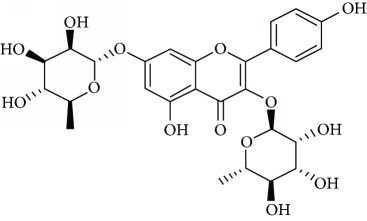 Kaempferitrin (10)	Oral 1, 5, 10, or 20 mg/Kg	Mice	FST, TST, and OFT	Reduced immobility time	Interaction with presynaptic 5-HT_1A_ receptors (serotonergic neurotransmitter system)	DR+	[74]
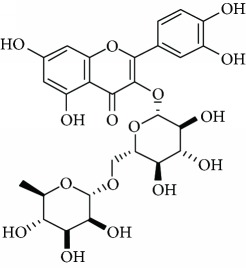 Rutin (11)	Oral 0.4, 4, 8, 16, and 32 mg/kg (fixed-ratio combination of rutin and hesperidin 71 : 21.5 *w*/*w*)	Mice	TST, OFT	No effects	Increased the 5-HT and DA levels in the CNS	DR+	[75]
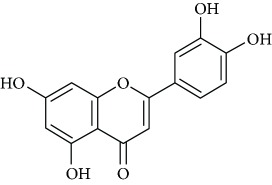 Luteolin (12)	Oral 50 mg/Kg	ICR mice	FST, TST	Reduced immobility time	Attenuated the expression of endoplasmic reticulum stress-related proteins in the hippocampus		[79]
	Oral 5 or 10 mg/Kg	ICR Mice	FST, OFT	Reduced immobility time	Potentiation the GABA_A_ receptor-Cl^−^ ion channel complex	DR+	[80]
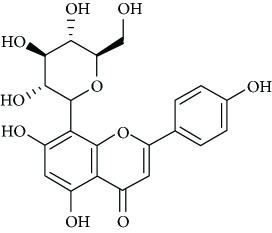 Vitexin (13)	Oral 10, 20, or 30 mg/Kg	Mice	MFTS, TST, and plus-maze	Reduced immobility time	Increased the catecholamine levels in the synaptic cleft Interactions with the serotonergic 5-HT_1A_, noradrenergic *α*2, and dopaminergic D1, D2, and D3 receptors	DR+	[81]
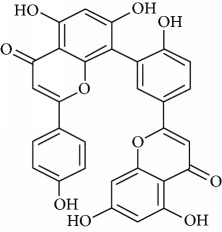 Amentoflavone (14)	Oral 6.25, 12.5, 25, or 50 mg/Kg	Mice	FTS, TST	Reduced immobility time	Interaction with serotonergic (5-HT_2_ receptors) and noradrenergic systems (*α*1-and *α*2-adrenoceptors)	DR+	[91]
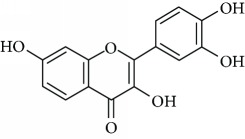 Fisetin (15)	Oral 10 or 20 mg/kg	Mouse	FST, TST	Reduced immobility time	Regulation of the central 5-HT in and NE, levels (inhibition of MAO activity)	DR+	[97]
	Oral 20, 40, or 80 mg/Kg	Mice	FST, TST	Reduced immobility time	Antagonized iNOS mRNA expression and nitrite levels via the modulation of NF-*κ*B Reversed LPS-induced overexpression of proinflammatory cytokine (IL-1*β*, IL-6, and TNF-*α*)	DR+	[98]
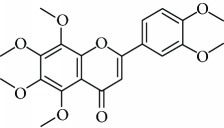 Nobiletin (16)	Oral 20, 50, or 100 mg/Kg	Mice	FST, TST	Reduced immobility time	Interaction with the serotonergic, noradrenergic and dopaminergic systems	DR+	[102]
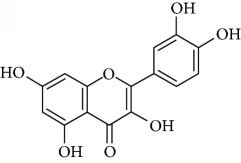 Quercetin (17)	Intraperitonial 50 or 100 mg/Kg	Rat	FST	Reduced immobility time	Attenuated depressive-like behaviours	DR+	[105]
	Oral 2.5, 5, or 10 mg/Kg	Rat	Acute stress, EPM	No effects	Protective effects on stress exposure	DR+	[106]
	Oral 20, 40, or 80 mg/Kg	Rat	OFT	Reduced immobility time	Neuroprotective effects Suppression of oxidative-nitrosative stress	DR+	[107]

FST: forced swimming test; CNS: central nervous system; EPM: elevated plus maze; SPT: sucrose preference test; BDNF: brain-derived neurotrophic factor; CMS: chronic mild stress; NGF: nerve growth factor; ERK: extracellular signal-related kinase; ST: splash test; MFTS: modified forced swimming test; NSFT: novelty-suppressed feeding test; NLRP3: nod-like receptor protein 3; TST: tail suspension test; GR: glucocorticoid receptor; OFT: open field test; CUMS: chronic unpredictable mild stress; DR^+^: dose/concentration response design; 5-HT: serotonin; DA: dopamine; NE: noradrenaline.
